# Health system redesign for maternal and newborn survival: rethinking care models to close the global equity gap

**DOI:** 10.1136/bmjgh-2020-002539

**Published:** 2020-10-14

**Authors:** Sanam Roder-DeWan, Kojo Nimako, Nana A Y Twum-Danso, Archana Amatya, Ana Langer, Margaret Kruk

**Affiliations:** 1 Health Section, UNICEF Tanzania, Dar es Salaam, Tanzania; 2 Global Health and Population, Harvard University T H Chan School of Public Health, Boston, Massachusetts, USA; 3 Maternal and Child Health, University of North Carolina at Chapel Hill Gillings School of Global Public Health, Chapel Hill, North Carolina, USA; 4 Health and Nutrition, Save the Children, Kathmandu, Nepal

**Keywords:** maternal health, health systems

## Abstract

Large disparities in maternal and neonatal mortality exist between low- and high-income countries. Mothers and babies continue to die at high rates in many countries despite substantial increases in facility birth. One reason for this may be the current design of health systems in most low-income countries where, unlike in high-income countries, a substantial proportion of births occur in primary care facilities that cannot offer definitive care for complications. We argue that the current inequity in care for childbirth is a global double standard that limits progress on maternal and newborn survival. We propose that health systems need to be redesigned to shift all deliveries to hospitals or other advanced care facilities to bring care in line with global best practice. Health system redesign will require investing in high-quality hospitals with excellent midwifery and obstetric care, boosting quality of primary care clinics for antenatal, postnatal, and newborn care, decreasing access and financial barriers, and mobilizing populations to demand high-quality care. Redesign is a structural reform that is contingent on political leadership that envisions a health system designed to deliver high-quality, respectful care to all women giving birth. Getting redesign right will require focused investments, local design and adaptation, and robust evaluation.

**Summary box**

- The dominant model of childbirth care in low-income countries today in which many women give birth in primary care facilities is not supported by accumulating global evidence and needs to be revised in order to address persistently high maternal and neonatal mortality rates.
- Health system redesign is a structural reform that enables all women to deliver in facilities with life-saving obstetric and newborn care; primary care clinics are reserved for high-quality antenatal and postnatal services.
- Health system redesign requires political leadership and policy change, hospitals that can deliver high-quality respectful childbirth care, primary care facilities that have mastered antenatal, postnatal and well-child care, health systems that decrease barriers to access, and populations that are empowered to demand high-quality care.
- A global double standard in childbirth care in which virtually all women in wealthy countries deliver in facilities with advanced obstetric and newborn care while women in low-income countries are asked to deliver in basic primary care facilities can no longer be tolerated.

## Introduction

Most of the world’s maternal and newborn deaths happen in low-income countries (LIC) where, despite substantial reductions, maternal mortality is 40 times higher and newborn mortality nine times higher than in high-income settings.^1 2^ Furthermore, a large rise in facility birth has not produced the expected outcomes; instead, maternal and neonatal mortality rates have plateaued in many LICs.^3–5^ One reason may be that one-third of facility births in LICs occur in basic primary care clinics where women and newborns have little recourse to lifesaving services in the event of a complication.^6^ This is the result of a two-tiered model of care that directs ‘low-risk’ women to primary care clinics and ‘high-risk’ women to hospitals despite accumulating evidence that primary care clinics cannot handle complications, referral systems do not function and risk cannot be accurately predicted. Moreover, recent expansions in infrastructure and roads have substantially improved access to hospitals. Redesigning health systems so that health services are provided by the right provider in the right place and at the right time was a key recommendation for improving quality of *The Lancet Global Health* Commission on High Quality Health Systems. Here we examine problems with the current approach, discuss the feasibility of redesign, propose reforms to transform current health systems, and argue that it is time to change policy and redesign health systems to provide high-quality services to all women and newborns.

## A global double standard in maternal and newborn care

Redesign is fundamentally a question of social justice; it seeks to restructure health systems so that all women, no matter where they live or their life circumstances, have ready access to advanced obstetric and neonatal care if a complication is to arise. This type of access is the norm in high-income countries (HIC) and middle-income countries where nearly all women deliver in facilities where they are attended by specially trained providers including physicians (eg, obstetricians, paediatricians or family physicians), nurses and midwives and have immediate access to emergency care.^6^ Many countries have further concentrated delivery care in high-volume hospitals because these can best maintain skills in treating complications. Studies estimate that less than 5% of women in HICs deliver at home where childbirth can be safe for select women if attended by skilled providers who can arrange rapid emergency transport to a nearby hospital.^6 7^ Risk stratification is used to move women with complicated pregnancies to higher level care, but even basic level 1 childbirth facilities in HICs are expected to have operating rooms, physicians trained in Caesarean section, blood banks and advanced newborn care.^8^

By contrast most LICs have adopted a two-tiered model of care in which high-risk women give birth in facilities with advanced obstetric and neonatal services and low-risk women give birth in primary care facilities that are expected to refer them to hospital if complications develop. No examples were found in the literature of HICs that use primary care clinics as the recommended level of care for low-risk women. As a result, one-third to one-half of facility births in the highest mortality countries occur in basic clinics without surgical or transfusion services. Most of these clinics have no physicians on staff and many have very low delivery volumes (<500 per year).^6^ These primary care clinics are more likely to be used by poor and remote women, worsening inequities in care.^9^ The end result is that increasing the number of women giving birth in facilities has not translated into the expected reductions in mortality.

**Figure 1:**
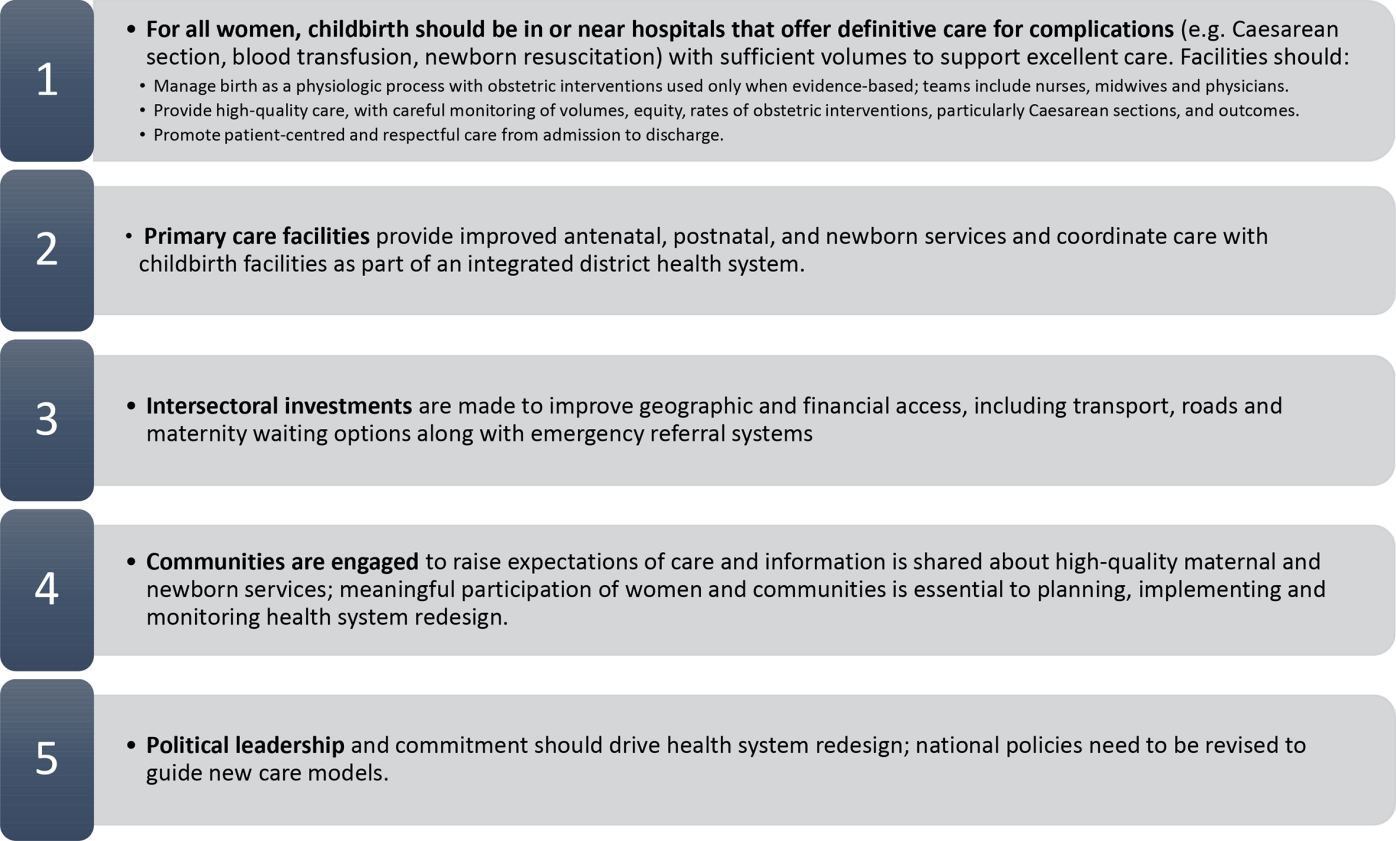
Core principles of health system redesign for maternal and newborn care.

We argue that this is a global double standard and that to reduce maternal and newborn mortality LIC health systems should be redesigned to bring them in line with global evidence and best practice.^10^ Redesign will not occur overnight, but if countries do not set course now for a future in which all women and babies have access to advanced maternal and newborn care in case of complications, then achieving equitable distribution of these services may become unreachable. Core principles of maternal and newborn health system redesign are summarised in figure 1.

## Revisiting the assumptions of the current model of care

Historically, the two-tiered obstetric care model for LICs was a well-intentioned response to high neonatal and maternal mortality in under-resourced health systems.^11^ It recommended that primary care clinics be used by low-risk women not only for antenatal and postnatal care, but also for childbirth. The goal was to offer essential healthcare to all people, consistent with the Alma Ata Declaration of 1978.^11^ Synthesising global experience and evidence from the past 20 years, we have identified five assumptions that undergird this model:

### Assumption 1: risk stratification in pregnancy can be used to select women for hospital care

Though risk stratification is a core function of high-quality antenatal care, many life-threatening complications first manifest at the time of delivery and cannot be predicted.^12 13^ Studies from HICs suggest that approximately 30% of women categorised as low-risk still develop complications and that available population risk models perform poorly in predicting outcomes or guiding treatment at the individual level.^14 15^ In the Netherlands more than half of nulliparous women and one in five multiparas starting labour in midwifery practices are referred to obstetric units despite being categorised as low-risk.^16^ Risk stratification is even more problematic in LICs where detectable antepartum risk factors such as multiple pregnancy, breech presentation, or pre-eclampsia are frequently missed due to poor-quality antenatal care.^10^ Furthermore, evidence shows that poor women are more likely to receive low-quality antenatal care that fails to deliver essential screening and treatment and that even with excellent antenatal care, it is impossible to predict all intrapartum complications.^12 13 17–20^ Risk stratification should be used to identify complicated pregnancies that require more specialised levels of care (eg, regional hospitals), but if 30% of those deemed low-risk still develop complications then it may not be adequate for selecting women to safely deliver remote to advanced care.

### Assumption 2: primary care clinics can provide good quality birth care

Basic emergency obstetric and newborn care facilities—primary care clinics with no surgical, blood transfusion or advanced neonatal services—have been deemed appropriate for low-risk birth in LICs. However, quality of maternal and neonatal care in primary care clinics has repeatedly been shown to be weak and access to delivery services in such settings is not associated with better outcomes when compared with giving birth at home.^21^ Furthermore, extensive efforts to improve quality of care in primary care clinics in LICs through coaching, feedback, checklists, decision support and technologies have been disappointing, with modest or null results in large-scale studies.^22 23^ Even if these strategies were effective, their scale-up across thousands of small facilities would be exceedingly difficult.

### Assumption 3: emergency referral is an effective response to complications

To save lives when obstetric and newborn complications arise, women should receive definitive treatment within 30 min or less.^24 25^ The standard approach if complications arise during childbirth in primary care settings is to refer women to hospitals. However, transporting acutely ill patients is challenging even in high-income settings with fully equipped advanced life support ambulances, reliable communication and good roads. Transport is only attempted if absolutely necessary and ideally, should occur before and not during delivery.^26 27^ In LICs, which lack these conditions, intrapartum transport is often lethal for mother and/or baby as is immediate postnatal transport of sick neonates with compromised cardiorespiratory function.^28 29^ Studies show that for women who develop complications, starting labour at a primary care clinic results in loss of precious time and increases the likelihood of maternal and perinatal mortality.^30–33^

### Assumption 4: pregnant women cannot get to hospital for delivery

While historically access to hospitals was limited in rural parts of LICs, rapid expansion in roads, transportation and health facility infrastructure has dramatically reduced travel time to hospital. A recent study in Haiti, Kenya, Malawi, Namibia, Nepal and Tanzania found that the vast majority of women now live within 2 hours of a hospital and that shifting all deliveries to hospital would reduce 2-hour access between 0.6% and 10%, depending on country.^34^ For many, though not all, women, hospitals are closer than 2 hours: the average travel time to hospitals in the study varied from 22 min in Haiti to 82 min in Tanzania. The finding is consistent with previous studies of access to advanced care.^9 35^ Moreover, LICs are rapidly urbanising, bringing advanced facilities within easier reach of the majority of women.^36^ This new demographic landscape is an opportunity for redesigning systems to equitably offer advanced care to all.

### Assumption 5: women prefer to give birth in nearby primary care clinics

While some women value local delivery, quality of care is more important than the proximity of the clinic for many women. Women routinely bypass local facilities for childbirth in search of higher quality care even if they incur additional costs.^37^ Several studies have found that wealthier women were more likely to bypass, exacerbating inequities in quality.^9 38 39^

## Benefits and risks of redesign

The primary benefit of redesign is improved health outcomes for all women and newborns.^5 6 10 21^ Health system redesign for childbirth services will most impact maternal deaths, intrapartum stillbirths and early newborn deaths; the latter comprise almost three-quarters of all newborn deaths, with about half of these early deaths occurring on the first day of life.^40^ It may also improve health system efficiency by concentrating obstetric and neonatal services and improvement efforts in fewer facilities. High delivery volumes allow providers to maintain skills for rare complications, to form multidisciplinary care teams, and also offers opportunities for continuous learning and for training new clinicians. Such environments may increase job satisfaction and motivation.^9 41–44^ Moving obstetric care from rural and isolated clinics to facilities in towns and cities may improve recruitment and retention of midwives and other health personnel.^45–47^ Finally, improving the quality of delivery care and surgical capacity may improve hospital quality more broadly.^48^ There are benefits for primary care too. A redesigned system reduces demands on primary care clinics, allowing them to focus on services that are their core competence including, for example, screening, prevention and management of the growing burden of non-communicable diseases.^49 50^ This in turn can decongest hospital outpatient clinics.

However, simply changing the location of childbirth in low-performing health systems without strengthening hospitals and implementing other reforms may have adverse consequences, especially for vulnerable populations.^51^ The quality and capacity of higher-level facilities in many LICs today is not adequate to produce excellent health outcomes.^10^ Shifting births to low-quality facilities may increase iatrogenic complications and, potentially, disrespectful care, especially where overcrowding occurs.^52^ It could inadvertently lead to overmedicalisation of birth and excessive Caesarean sections. Shifting births to advanced facilities may reduce access to advanced obstetric and neonatal care for women in rural and remote areas. This may lead to increasing gaps between socioeconomic groups and exacerbate the urban–rural divide in access to essential social services.^53 54^ Finally, weak health systems may struggle with the ‘capability trap’: donor and political pressure to rapidly appear capable, despite a lack of capacity to implement policy and programmes.^55^ Relabelled but not redesigned systems will not achieve improvements in maternal and neonatal health.

## Implementing redesign to achieve high quality, respectful care

To maximise the above gains and guard against the risks, each country will need to develop a locally specific model for redesign, based on a thorough assessment of health needs and health system assets. While a policy of redesign may be national, given subnational variations in health systems, population density, geography and socioeconomic factors, the unit of planning and implementation may need to be more local. For example, redesign in a densely populated urban area with many facilities offering childbirth services may not need to improve transport, while the plan for a rural area that experiences seasonal isolation due to flooding may be highly dependent on better transport infrastructure. The smallest unit for service delivery redesign planning is a network of facilities including an advanced obstetric and newborn care facility (a hub) and linked primary care facilities (spokes). Successful redesign efforts will require input from a range of stakeholders, including health system and other sector managers, public and private providers and healthcare users, especially more vulnerable and remote populations. A systems thinking approach that anticipates the non-linear and system-level effects of the change will be vital to success.^56^ Planners will need to monitor the ‘inputs, outputs, initial, intermediate and eventual outcomes, and feedback, processes, flows, control and contexts’ of service delivery redesign.^56^ Careful monitoring for changes in delivery volumes, unnecessary clinical interventions (ie, Caesarean section) and socioeconomic signals of inequitable service provision will be especially important. Later we outline five key elements required for implementation of health system redesign. Proper sequencing is essential: policy change should only be implemented after hospitals, clinics and transport systems have been improved.

### Strengthen lifesaving, respectful care in hospitals

For the majority of women, midwife-led care, supported by physicians with obstetric training, leads to good health outcomes, avoids over-intervention and creates clinical environments that respect women and promote their agency.^57–59^ In the context of redesign, both onsite and near-site midwife-led birth units can make woman-centred care possible while ensuring immediate access to emergency obstetric intervention in case of complications (see table 1). However, in order for midwifery to meet its potential as the cornerstone of a redesigned health system, current gaps in the preservice and in-service education of this cadre need urgent attention.^60 61^ This is especially important as facility childbirth rates rise and as evidence of disrespectful treatment of labouring women is growing.^52 62–64^ International guidelines suggest that midwifery education should take a human-rights approach that produces clinicians who are competent to support physiological childbirth for women of all backgrounds, to empower them through the continuum of care, to learn and use evidence and to respond quickly to complications and refer to obstetric colleagues.^65^

**Table 1:** Models of midwife-led delivery care with rapid access to advanced care

Location	Programme description	Results	Study notes
**Onsite midwife-led birth unit (OMBU)—low-risk birthing unit on the same premises as, but separated from, an obstetric unit with capacity to provide care for severe peripartum complications**			
South Africa^19^	OMBU is in the same facility as obstetrics (OB) unit. Clinical interventions are kept to a minimum, but midwives can provide opioid injections, artificial rupture of membranes, electronic fetal monitoring. Care is provided based on the prevailing primary care guidelines and is administered and funded by the primary care service, rather than the hospital.	Facility deliveries increased from 6352 to 7375 per year and Caesarean section (CS) rates were reduced from 38% to 35%.	Routinely collected data from 12 months before and after implementation of OMBU (2011–2013).
China^83^	Midwife-led unit for low-risk clients. Located in a hospital and close to the standard OB unit. Provides home-like environment for childbirth, where women can move about freely, birth companionship is encouraged, and interventions are kept to a minimum. Complications are referred to the standard OB unit.	CS rate was 8.4% in the OMBU vs 38.5% in the standard care unit, with lower rates of oxytocic augmentation 15.5% in OMBU (15.5% vs 39.8%). Most (94%) of OMBU clients reported being happy with their birth experience in the OMBU.	Retrospective study of the first 6 months of the implementation of the OMBU, involving 452 women (2008).
Hong Kong^84^	OMBU is in the same unit as OB and uses the same protocols. Midwives manage all aspects of care and decide if and when to consult OB.	Lower obstetric intervention rates but no difference in 5 min APGAR scores less than seven and no difference in transfers on account of fetal distress.	Randomised controlled trial with 1050 low-risk women (1994–1995).
Norway^85^	OMBU is on the same floor as OB unit and provides a home-like environment that minimises interventions. No inductions or augmentation of labour in OMBU. Midwives manage all aspects of intrapartum and postpartum care and consult OB if complications arise.	No difference in low 5 min APGAR scores, transfers to neonatal intensive care unit or CS rates.	Prospective cohort study of 453 primiparous low-risk clients conducted (2001–2002).
Japan^86^	OMBU is on the same premises as the OB unit and provides a home-like environment in traditional Japanese rooms. Midwives refer any complications to OB and interventions are limited.	No difference in obstetric complications (postpartum haemorrhage or 3rd/4th degree perineal tears) or CS rates. No difference in neonatal outcomes (5 min APGAR score less than 7 or umbilical artery pH).	Retrospective study of 1031 low-risk women (2008–2010).
**Near-site midwife-led birth unit (NMBU)—low-risk birthing unit located outside of, but close to (and contractually linked) to an obstetric unit with capacity to provide care for severe peripartum complications**			
USA^87^	NMBU across the street from a rural referral hospital with which it partners. NMBU was set-up by the referral hospital to provide care for indigent rural population. Midwives manage all low-risk antenatal care and deliveries at the NMBU; family physicians manage high-risk clients, medical problems, complicated deliveries and provide paediatric care; and OBs consult on particularly high-risk clients and perform CS. Outreach visits are made to counties where there is no health centre.	Facility deliveries increased by 30% over 5 years with the introduction of the maternity clinic with lower costs in the NMBU than in the obstetrician-led practice. There was no significant change in newborns requiring specialist care.	Before and after review (1984–1989).
Nepal^88^	NMBU is attached to a hospital with OB services. Labour management guided by clearly defined labour ward protocols. Discharge from unit occurs within 1 day, with appropriate counselling.	NMBU clients had lower rates of interventions, including CS. For normal births, delivery at the NMBU cost $11 vs $27 for standard care.	Cohort study of 988 low-risk women (1997–1998).

High hospital maternal and neonatal case fatality rates and perioperative mortality rates in LICs also point to needed improvement in surgical services and neonatal care for sick and premature newborns.^66–69^ Redesigned hospitals will require neonatal care units with sufficient supply of equipment, supplies, medication and human resources.^70^ These units should be physically separated from other patient care areas, be staffed by providers with training on the care of sick and premature neonates and be able to accommodate and engage families.^71^ In countries where specialists are in short supply or concentrated in urban areas, telemedicine and telementoring options will need to be explored.

Curriculum reform and twinning relationships between well-performing facilities and local facilities can raise quality standards across these disciplines and interactive, in-service training, including simulation, can be used to refresh infrequently used skills alongside coaching and mentoring.^72^

### Boost primary care

As part of service delivery redesign, health systems will need to clearly articulate the core services best offered in primary care facilities and elevate primary care such that it is recognised as an area of expertise, not a minimalist version of hospital care. Primary care clinics will need to improve the provision of evidence-based antenatal and postpartum services, as well as maintain registries of pregnant women, work with women and couples on birth planning and coordinate care with higher-level facilities. One area for urgent improvement is detection and mitigation of maternal and fetal risk, including anaemia, malaria, HIV, multiple pregnancy and the like. Primary care, through community health worker programmes, is also best placed to follow-up with the mother–baby dyad in the community after discharge, especially in settings where short postpartum hospital stays are the norm.^73^ From the patient’s perspective, primary care should be the nucleus of their maternal and newborn health services.

In a redesigned health system, primary care facilities providing antenatal care should be linked to an advanced care facility to allow for sharing of care across the continuum and efficient communication. Further, advanced care facilities and their linked primary care centres could create learning collaboratives that meet regularly to review complex cases and solve problems.^74^

### Promote equitable access to care

Women who live far from a hospital will need to travel before the onset of or during early labour. Some remote areas may require upgrading health centres to provide advanced care, new roads and bridges, more reliable transportation options and/or patient-centred maternity waiting homes.^75–78^ Financial and other socioeconomic barriers must also be addressed, for example, through vouchers, cash transfers and comprehensive health insurance benefits. Novel solutions, including ride-sharing and Airbnb-like or other maternity home waiting options, need to be tested (see table 2). Without fully integrating geographic access interventions, redesign could exacerbate childbirth disparities and leave the most remote and vulnerable families behind.^79^ Although intrapartum referral will be needed less under redesign, emergency referral systems will need to be strengthened for transport of women and newborns with severe complications to specialised facilities.

**Table 2:** Options for improving geographic access to hospitals

Category	Option	Details/examples
Infrastructure	Develop additional advanced neonatal and obstetric capacity	In areas with no access to hospitals or other facilities providing advanced care (surgery, newborn intensive care) within 2 hours, such facilities could be established, or existing facilities could be upgraded. This must be done equitably, preferably using geographic mapping and population density analyses. In Tanzania, health centres are being upgraded for surgical capacity to increase access to surgical care across the country.^75 89^
	Construct roads, bridges and other physical connections	Extending road networks to rural communities, constructing bridges and providing ferries and other physical infrastructure to connect communities are means to reduce the time and distance to reach care. Bangladesh added over 50 000 km of roads and 300 km of bridges to the transportation network between 2001 and 2010, decreasing travel time and increasing access to facilities, which likely contributed to the reductions in maternal mortality observed in that period.^90^ A similar attribution is made for Cambodia.^91^
Transportation and referral	Expand use of public transportation and private vehicles	In many communities, public transportation options are available and predictable. Once women plan to reach delivery care early, these public buses, trains and share taxis can offer an affordable and reliable means of transportation. On-demand private taxis or community-owned vehicles are also a viable means of transportation for both rural and urban populations.
	Use ride-share technologies	As mobile penetration increases in low-income settings, ride-share is becoming increasingly popular, and this technology can be used in facilitating maternal transportation. An uber-like application piloted in Homa Bay County in Kenya was found to provide 1 hour access to skilled birth care to nearly 90% of users.^92^
	Mobilise community transportation funds	Community funds to cover emergency transportation have been used in a variety of locations. For example, Health and Insurance Management Services Organisation trains communities to manage their own low-cost emergency transportation fund in rural Tanzania.
	Provide dedicated medical transportation	When primary care centres have dedicated vehicles for medical transportation, reaching advanced care is easier and/or safer for patients. In rural Ghana, the provision of modified three-wheeled motorcycles to health centres was found to have resulted in a shifting of deliveries from primary care to advanced facilities.^93^
	Improved communication	New digital technologies and expanded mobile telephone and internet coverage mean that communication between facilities can improve. For example, WhatsApp is being used in rural Tanzania to ‘give report’ between referring and receiving facilities.
Waiting options	Establish dignified maternity waiting homes	Maternity waiting homes enable women who are very remotely located to stay in or close to a health facility when they are near term in order to be close to care when they go into labour. A recent study in Ethiopia found that hospitals with maternity waiting homes had 40%–50% lower rates of maternal and perinatal complications compared with hospitals without waiting options.^94^
	Encourage staying with relatives in towns with advanced obstetric and neonatal care during last few weeks of pregnancy	With increasing urbanisation throughout the world, including in low-income and lower middle-income countries, an increasing proportion of rural residents will have relatives living in urban and peri-urban areas where health facilities with advanced obstetric and neonatal care are likely to be found. Thus, encouraging pregnant women living in rural areas to temporarily stay with relatives in towns may be preferable than maternity waiting homes for some.
	Explore Airbnb-like options	Where there are no maternity waiting homes, lodging with a host can bring women closer to advanced care when they are near term. An Airbnb-like online platform would allow clients to select options that meet their specific needs (eg, hosting siblings or birth companions) and rate their lodging experience. This platform can be used to plan the stay during antenatal care and the rating function provides an important accountability mechanism. This initiative could be combined with a voucher scheme that defrays the cost of stay for the woman.
Financing mechanisms	Institute conditional cash transfer schemes for delivery in advanced facilities	Making monetary payments to women who deliver in advanced facilities can provide an incentive for women to continue to do so. Evidence from India’s Janani Suraksha Yojana programme and from studies in sub-Saharan Africa suggests that conditional cash transfers are a viable demand-side strategy to increase access to services and bridge equity gaps, but only if facilities are of adequate quality.^95 96^
	Provide vouchers for facility deliveries and/or transport	Voucher programmes can reduce or remove the cost of reaching and obtaining quality delivery care. Voucher programmes have enabled women in rural Uganda to access private transportation options during labour without any upfront costs and helped subsidise maternal care services for poor women in Kenya.^97 98^ These schemes can be further targeted for delivery in advanced facilities.

### Ignite demand and construct accountability channels

In addition to lowering barriers to access, health system leaders will need to raise demand for higher-level maternity care by informing women and families of the rationale and specifics of redesign and by raising their expectations of delivery and newborn care.^10 80^ Women, families and communities will need to understand what high-quality care for childbirth means and where this care can be received. Community outreach and social marketing through radio, television, and community drama can be effective means of educating the community and increasing demand for quality. Participatory methods, including women’s groups practicing learning and action cycles, are also a good way to meaningfully engage people in improving systems.^81^ These efforts should include the whole family, not just women, as well as key social and religious leaders who may be influential. In many countries, providing health services close to communities is politically important, and communicating the rationale for removing delivery services from primary care clinics will be essential. Redesign should be accompanied by means for feedback and redress at all levels of the system, for example, through dedicated phone lines or text systems. More research is needed to understand how best to improve accountability and share information about quality of care with communities.^82^

### Political leadership and policy change

Service delivery redesign is fundamentally a political choice and must be led by political leaders who believe that a double standard for women and newborns in LICs is no longer acceptable. Political commitment is central to determining whether redesign is realistic and how quickly it can be implemented. Though a daunting task, we are already seeing such commitment from some countries. In Kakamega County, in Kenya, the county government has committed to implementing redesign; this is after a feasibility assessment was conducted to determine readiness for redesign. The government is now moving into a planning phase where strategies will be developed to close the identified gaps and ensure that no woman is left behind. The government also plans to implement redesign in a phased manner to allow for adaptation as necessary. Policy review and change will be necessary in many countries where current guidelines and policies reflect the assumptions underlying the two-tiered model for LICs.

## Conclusion

Service delivery redesign is not a single intervention nor is there a single model—it is a structural health system reform that if implemented correctly will save lives. Redesign will not be easy to implement nor will results be seen quickly. Successful redesign will require systems thinking, political leadership, locally specific solutions, money, skilled providers and time. This may all seem impossible, but the alternative, to allow remote and vulnerable families to receive care in facilities that cannot handle complications and are too far from advanced care to make referral possible, is simply not acceptable. Negative consequences are possible if all births are shifted to hospitals without investing in quality, access and accountability to communities. These negative externalities must be anticipated and mitigated.

Given the scale and ambition of redesign and its potential to guide future health systems, research should be a central component of all redesign efforts. Preparating for redesign includes systematic feasibility analyses that map the health system network and capacity, supply of health workers, care quality, transportation systems and community preferences and utilisation patterns. Prospective, rigorous evaluation is required to assess impact and costs, adapt programmatic interventions and promote the spread of best models. Programme data will need to be carefully disaggregated and analysed to capture the experience and outcomes of vulnerable populations. Implementation science methods that include qualitative research will be needed to capture outcomes, implementation fidelity, patient voice and unintended consequences.

Health system redesign is intentionally ambitious; nothing short of large-scale changes will close the large global equity gap in access to high-quality care. We have long known that childbirth in advanced facilities with high-quality obstetric and neonatal care is the strategy that saves the most lives, but this has been considered out-of-reach for most women in LICs. It is time to shift course and make lifesaving, respectful care the standard of care for all women.
